# Integrated Application of Selenium and Silicon Enhances Growth and Anatomical Structure, Antioxidant Defense System and Yield of Wheat Grown in Salt-Stressed Soil

**DOI:** 10.3390/plants10061040

**Published:** 2021-05-21

**Authors:** Ragab S. Taha, Mahmoud F. Seleiman, Ashwag Shami, Bushra Ahmed Alhammad, Ayman H. A. Mahdi

**Affiliations:** 1 Botany Department, Faculty of Agriculture, Beni-Suef University, Beni Suef 62521, Egypt; ragab.salama@agr.bsu.edu.eg; 2 Plant Production Department, College of Food and Agriculture Sciences, King Saud University, P.O. Box 2460, Riyadh 11451, Saudi Arabia; mseleiman@ksu.edu.sa; 3 Department of Crop Sciences, Faculty of Agriculture, Menoufia University, Shibin El-Kom 32514, Egypt; 4 Biology Department, College of Sciences, Princess Nourah bint Abdulrahman University, Riyadh 11617, Saudi Arabia; 5 Biology Department, College of Science and Humanity Studies, Prince Sattam Bin Abdulaziz University, Al Kharj Box 292, Riyadh 11942, Saudi Arabia; b.alhammad@psau.edu.sa; 6 Agronomy Department, Faculty of Agriculture, Beni-Suef University, Beni Suef 62521, Egypt; drayman.hamdy@agr.bsu.edu.eg

**Keywords:** wheat, selenium, silicon, salinity, yield, anatomy, antioxidants

## Abstract

Selenium (Se) and silicon (Si) are considered advantageous elements to induce plants’ tolerance to various environmental stresses. Wheat yield is negatively affected by salinity stress, especially in dry and semi-dry areas. Therefore, the objective of the current study was to investigate the effects of Se, Si and their combinations (0 as control, Se_15_*,* Se_30_*,* Si_15_*,* Si_30_*,* Se_15_ + Si_15_*,* and Se_30_ + Si_30_ mM) in alleviating the deleterious effects of salinity stress (7.61 dS m^−1^, real field conditions) on anatomical characteristics as well as the physio-biochemical and productivity parameters of wheat plants. The selenium and silicon treatments and their combinations caused significant amelioration in growth, anatomical and physiological attributes, and grain yields of salinity-stressed wheat in comparison with the untreated plants (control treatment). The integrated application of Se_30_ + Si_30_ significantly increased plant growth (i.e., plant height 28.24%, number of tillers m^−2^ 76.81%, fresh weight plant^−1^ 80.66%, and dry weight plant^−1^ 79.65%), Fv/Fm (44.78%), performance index (PI; 60.45%), membrane stability index (MSI; 36.39%), relative water content (RWC; 29.39%), total soluble sugars (TSS; 53.38%), proline (33.74%), enzymatic antioxidants (i.e., CAT activity by 14.45%, GR activity by 67.5%, SOD activity by 35.37% and APX activity by 39.25%) and non-enzymatic antioxidants (i.e., GSH content by 117.5%, AsA content by 52.32%), yield and its components (i.e., number of spikelets spike^−1^ 29.55%, 1000-grain weight 48.73% and grain yield ha^−1^ 26.44%). The anatomical traits of stem and leaves were improved in wheat plants treated with Se_30_ + Si_30_. These changes resulting from the exogenous applications of Se, Si or their combinations, in turn, make these elements prospective in helping wheat plants to acclimate successfully to saline soil.

## 1. Introduction

Wheat (*Triticum aestivum* L.) is considered the most important cereal crop worldwide that is sown for its grains as an essential food. Globally, the cultivated area of wheat in 2017 was about 219 million hectares, with a total production of 772 million tons of grain [1]. In addition, its grains contain a high percentage of carbohydrates and protein [2,3,4]. Wheat is considered a major source of protein (about 13%) in human food worldwide and it contains mineral nutrients and dietary fiber [4]. Roughly 85% of world habitants derive most of their calories and protein from wheat grains [5,6]. The demand for wheat grains is increasing as a result of the increase in world inhabitants [2,4]. However, wheat grain productivity is significantly influenced by environmental stresses, including salinity and nutrient deficiency, which can cause osmotic and drought stresses [2,7,8,9,10,11,12].

Salinity is one of the most critical abiotic stresses; it is responsible for reductions in the productivity of different crops [3,13,14]. Almost one-third of cultivated land is negatively influenced by salinity stress, which presents a serious menace to the availability of cultivated lands and human food. Despite significant efforts, there has been little success in raising salt-tolerant wheat genotypes [15]. This is due to salinity tolerance and the complex nature of plant-specific morphological, physiological and metabolic processes that regulate the mechanisms of salt tolerance [16,17]. Salinity stress can lead to a functional disturbance in plant cells and antioxidant defenses because of reactive oxygen species (ROS) extravagant production of ^1^O_2_, O_2_, H_2_O_2_, and OH^−^, in addition to the disturbances of cell membranes and lipid peroxidation that result from the stress of Na^+^ ions and ROS [14,18,19]. As result of increasing world population, finding methods and strategies that can mitigate salinity stress on crop yield production are considered of utmost importance. Some strategies have been applied to plants to overcome the stress of soil salinity. Exogenous application of Se, Si and their combinations can be a suitable option to mitigate the negative effects of salt stress.

Selenium (Se) is a useful element for plants [20] as it can play an important role in delaying the plant senescence [21,22] and enhancing its growth [23]. Additionally, Se alleviates unfavorable phenomena resulting from different environmental stresses such as heavy metals [24], cold stress [25], heat stress [26,27], drought [28,29] and salt stress [30,31]. Moreover, Se boosts mechanisms of potential defensive against stress, including the stimulation of antioxidant enzymatic and non-enzymatic activities (i.e., antioxidant system); consequently, it can lessen stress-induced oxidation [25]. In addition, Se can ameliorate photosystem efficiency II (PSII), promote fluorescence of chlorophyll and decrease the disintegration of chlorophyll [25]. Furthermore, it can contribute to the regulation of plant water status via boosting the absorption efficiency of water and reducing the water loss through plant tissue [32].

Silicon (Si) is one of the micronutrients that can promote plant tolerance against various environmental stresses, including salinity [33,34,35]. Despite the abundance of Si in the soil, most Si is formed as silicon dioxide (SiO_2_), which is not directly available for plant uptake. Si can improve plant growth, increase crop productivity, enhance photosynthesis efficiency and nitrogen fixation, and ameliorate antioxidant defense systems against various environmental stresses [36,37,38,39]. Moreover, the accumulation of Si in plant tissues can stimulate the production of phenolics and phytoalexins and can increase pathogen resistance [40].

As mentioned above, the current work was conducted to investigate the defensive role of Se, Si and their combinations as exogenous applications to mitigate the negative impacts of salinity stress through enhancing physiological and biochemical traits as well as enhancing the activities of antioxidant defense systems in wheat plants. Furthermore, this study investigates the possible amelioration in growth and production of wheat crops under real conditions of salinity stress through the exogenous application of Se and/or Si.

## 2. Material and Methods

### 2.1. Study Location, Experimental Design and Treatments

Two field trials were run at the Experimental Farm, Agriculture Faculty, Fayoum University, Egypt (29°17′ N; 30°53′ E), during the two successive winter seasons of 2018/2019 and 2019/2020 in order to study the individual and combined influence of Se and Si on growth, anatomical structure, physio-biochemical, enzymatic antioxidants and yield of wheat plants grown in actual saline soil (7.61 dS m^−1^). The experimental areas were divided into plots with a size of 12 m^2^ (3 m × 4 m). Rows were 4 m long and spaced 15 cm apart. Healthy and uniform wheat grains (Misr3 cultivar) were attained from the Field Crop Research Institute, Giza, Egypt, and were sown at a seeding rate of 153 kg ha^−1^ on 17 and 19 November 2018 and 2019. All other agricultural practices were done as recommended for wheat yield trial packages. The synthetic fertilizers were added at the rate of 200 kg N ha^−1^, 55 kg P_2_O_5_ ha^−1^ and 60 kg K_2_O ha^−1^. The P and K fertilizers were added during land preparation, while N fertilizer was divided into three doses (20% at sowing, 40% before first irrigation and 40% before second irrigation). The wheat plants were irrigated 6 times during the growing seasons, with 15–20 days between each of the two irrigations, depending on the precipitation rate.

The experimental design in the current study was a randomized complete block design with four replications. The wheat plants were sprayed with only tap water for the control (0 mM) and 15 mM Se, 30 mM Se, 15 mM Si, 30 mM Si, 15 mM Se + 15 mM Si and 30 mM Se + 30 mM Si at leaf development (BBCH stage 13) and tillering stages (BBCH stage 23) [41]. The concentrations of Se and Si were determined based on a preliminary study in a glasshouse. The forms of Se and Si were sodium selenite (Na_2_SeO_4_) and sodium silicate (Na_2_SiO_3_), respectively. The exogenous application of Se and Si treatments was used instead of soil application due to its efficient effect and the small applied quantities.

The physico-chemical properties of the experimental soils were analyzed according to Dahnke and Whitney [42], Jackson [43] and Chapman and Pratt [44] and are presented in Table 1. The average values of soil analysis during growing seasons were pH, 7.38; EC, 7.61 dS m^−1^; organic matter, 0.92%; total N, 0.068%; available P, 7.51 mg kg^−1^; available K, 177 mg kg^−1^ (Table 1). The weather conditions, including temperatures, precipitation and relative humidity, during the two growing seasons in the region of the experiments are shown in Table S1.

### 2.2. Measurements

#### 2.2.1. Growth and Yield Measurements

At 50% of heading stage (BBCH 55 stage), wheat plants (*n* = 9) were carefully collected from each experimental plot and dipped in a bucket of water. Plants were gently shaken to remove all adhering soil particles. Then, plant height was measured using a meter scale. The number of tillers per m^2^ was counted. The plants were weighed for fresh weights and then placed in the oven at 80 °C for 48 h. The dried plants were weighed to record their dry weight. At harvest, wheat plants within each plot were harvested, and all yield traits, i.e., number of spikes per m^2^, number of spikelets per spike, spike length, 1000-grain weight and grain yield per hectare were measured.

#### 2.2.2. Stem and Leaf Anatomy

Anatomy samples, including flag leaf and stem, were taken from each experimental plot at 50% of heading stage (BBCH 55 stage) [45]. The leaf and stem samples were fixed in FAA solution at a rate of 50:35:10:5; ethyl alcohol: distilled water: formalin: glacial acetic acid, respectively. The dehydration was done in tertiary butyl alcohol series. Paraffin wax was used for embedding the samples at 54–56 °C m.p.; then, the samples were cut into 20 μ thickness by microtome and adhered by Haupt’s adhesive. Crystal violet erythrosine combination was used for staining the sections [45]. Finally, the average of the nine readings was recorded using a micrometer eyepiece.

#### 2.2.3. Physiological Traits

Chlorophyll fluorescence was analyzed on two different days at 50% heading (BBCH stage 55) [41] using a portable fluorometer (Handy PEA, Hansatech Instruments Ltd., Kings Lynn, UK). Integration with Hansatech dark-adapted leaf clips was used for rapid screening. Fv/Fm value was observed after 20 min. Saturating pulse was applied, typically 0.8 s at an intensity of the light beam of 4000 μmol m^−2^ s^−1^. On each day, the top three fully expanded leaves were randomly selected from different three plants within each experimental plot. Fluorescence traits were conducted using the maximum quantum yield of PS II Fv/Fm as described in the following equation [46]:

$$F_{v}/F_{m}\;=\;\left(F_{m}-F_{0}\right)/F_{m}$$

Performance index (PI) of the photosynthesis based on the equal absorption (PIABS) was determined as described by Clark et al. [47].

Membrane stability indices (MSI) were assessed from nine samples for each treatment using duplicate 200 mg samples of fully expanded leaf tissue [48]. The first sample of the duplicate samples was added to 10 mL of double-distilled water in a test tube, which was then boiled at 40 °C for 30 min in a water bath. The electrical conductivity (C1) of the solution was measured via a conductivity bridge. The second sample of the duplicate samples was heated at 100 °C for 10 min, and then the conductivity was measured (C2). Finally, the following equation was used for calculating MSI:

$$MSI\;\left(\%\right)\;=\;\left[1-\left(C1\;÷\;C2\right)\right]\;\times \;100$$

Excluding the midrib, 2 cm diameter fully expanded fresh leaf discs were collected to measure the relative water contents (RWC) [49]. The discs were weighed as fresh weight (FW) and were instantaneously floated on double-distilled water for 24 h in Petri dishes under dark conditions for the leaves’ saturation with water. Any holding water was blotted dry, and the turgid weight (TW) was determined. Finally, the leaf discs were placed in the oven for 48 h at 70 °C for determining the dry weight (DW). The RWC was then calculated with the following equation:

$$RWC\;\left(\%\right)\;=\;\left[\left(FM-DM\right)\;÷\;\left(TM-DM\right)\right]\;\times \;100$$

##### Non-Enzymatic Antioxidant

The glutathione (GSH) content in the fresh leaf tissue of wheat was analyzed as described by [50]. Fresh wheat leaf tissues were ground in a meta-phosphoric acid (2%) and afterward centrifuged at 17,000× *g* for 10 min to be homogenized. Sodium citrate was added for neutralizing the supernatants and each assay. The solution was formed from 700 μL of 0.3 mM NADPH, 100 μL distilled water, 100 μL of 6 mM, and 50-dithiobis-2-nitrobenzoic acid. About 100 μL of the extraction was stabilized at 25 °C for 4 min. Finally, 10 μL of GSH reductase (50-unit mL^−1^) was inserted and mixed with the extraction, and then the readings were recorded at 412 nm using a spectrophotometer.

Ascorbic acid (AsA) was analyzed as explained by [51]. A 2 mL sample of 2% dinitrophenylhydrazine was mixed with the extracted samples in a 6% trichloroacetic acid (TCA). Afterwards, a drop of thiourea (10%) in an ethanol (70%) was added into the mixture. Thereafter, the mixture was immersed in a boiling water bath for 15 min. Finally, the mixture was cooled in 5 mL of H_2_SO_4_ (80%) at 0 °C and the absorbance was read using the spectrophotometer at 530 nm.

To analyze the total soluble sugars (TSS), the alcohol extract was prepared as explained by [52] A total of 3 mL of anthrone reagent, 150 mg of anthrone in 100 mL of 72% sulphuric acid, was mixed with 0.1 mL of the alcohol extract. This solution was boiled for 10 min and then was left for cooling. Finally, the absorbance was measured using the spectrophotometer at 625 nm.

Free proline (mg g^−1^ DW) was analyzed as described by [53]. About 500 mg of dried wheat leaves was milled in sulfosalicylic acid (3%) and then was centrifuged for 10 min at 10,000× *g*. The supernatants were mixed with ninhydrin (2%) and glacial acetic acid. Then, the mixture was heated for 30 min at 90 °C. Next, it was left for cooling at room temperature. The toluene was added to the previous cooled mixture, and the absorbance was measured using the spectrophotometer at 520 nm.

##### Enzymatic Antioxidants

Catalase (CAT) activity was analyzed by determining the H_2_O_2_ consumption as described by [54]. The reaction mixture involved a 25 mM Tris-acetate buffer, pH 7.0, 20 mM H_2_O_2_ and 0.8 mM Na-EDTA. The activity of catalase was implemented at 25 °C using a spectrophotometer to read the absorbance at 290 nm, and it was expressed as A_290_ min^−1^ g^−1^ protein.

Glutathione reductase activity (GR) was analyzed according to [55]. The activity of this antioxidant was measured after monitoring the NADPH’s oxidation for three absorbances read at 340 nm using aspectrophotometer, and it was expressed as A_340_ min^−1^ mg^−1^ protein.

Superoxide dismutase activity (SOD) was evaluated according to [56,57,58] by monitoring the inhibition of the photochemical reduction of nitro blue tetrazolium (NBT). The unit of SOD activity was determined as the amount of the required enzyme for the reduction of 50% NBT and was expressed as A_564_ min^−1^ g^−1^ protein.

Ascorbate peroxidase activity (APX) was estimated following the technique published in [55]. The activity was measured by recording the optical density using a spectrophotometer at 290 nm and was expressed as A_290_ min^−1^ g^−1^ protein.

### 2.3. Statistical Analysis

The data obtained from the effects of Se, Si and their combinations on the physiological and biochemical traits and productivity of wheat grown under salinity stress were subjected to analysis of variance (ANOVA) after testing the homogeneity of the error variances. A combined analysis of the data obtained from the two seasons was done, and the significant differences among different treatments were compared at *p* ≤ 0.05 using Duncan’s multiple range test.

## 3. Results

### 3.1. Growth Traits of Triticum Aestivum

**Figure 1:**
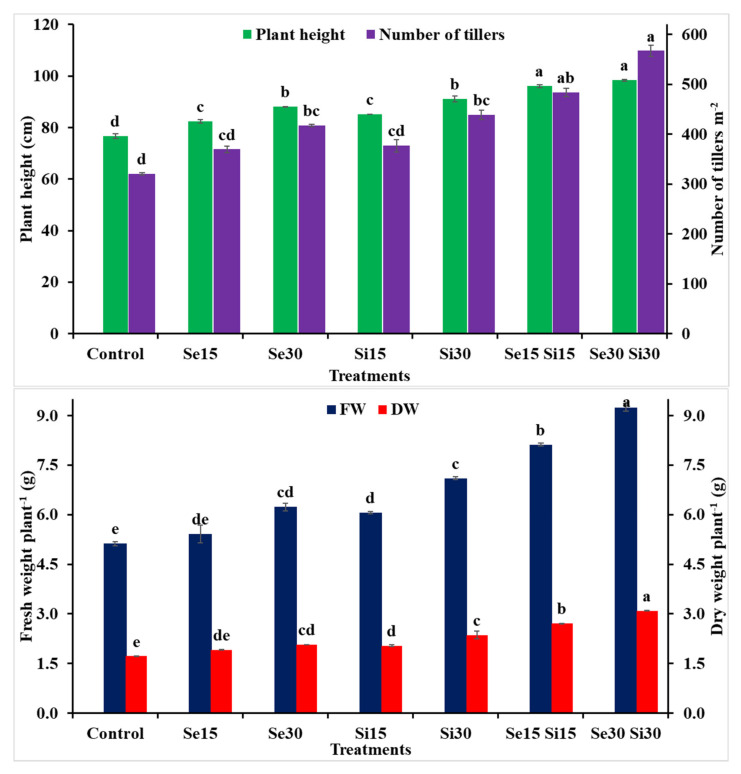
Exogenous application effect of selenium (Se), silicon (Si) and their combinations on plant height and number of tillers (m^−2^) (upper graph); fresh and dry weight (lower graph) of wheat plants grown in saline soil. Columns of each traits with the same letters are not significantly different at *p* ≤ 0.05.

As shown in Figure 1, growth traits (i.e., plant height, number of tillers (m^−2^), fresh and dry weight plant^−1^) were negatively affected in wheat plants grown under real saline soil conditions (control). However, the salt-stressed plants treated with selenium (Se), silicon (Si) and/or their combinations showed significant improvements in the above mentioned growth traits compared to untreated stressed plants (Figure 1). The maximum value was recorded with Si_30_ in combination with Se_30_ followed by the treatment with Si_15_ in combination Se_15_, as compared to other treatments. Increases in growth traits were recorded for plant height (28.24 and 25.24%), number of tillers per m^−2^ (76.81 and 50.87%), fresh weight plant^−1^ (80.66 and 58.59%), and dry weight plant^−1^ (79.65 and 57.56%), for Se_30_ + Si_30_ and Se_15_ + Si_15_, respectively, compared to the control treatment.

### 3.2. Stem and Leaf Anatomical Structure of Triticum Aestivum

**Figure 2:**
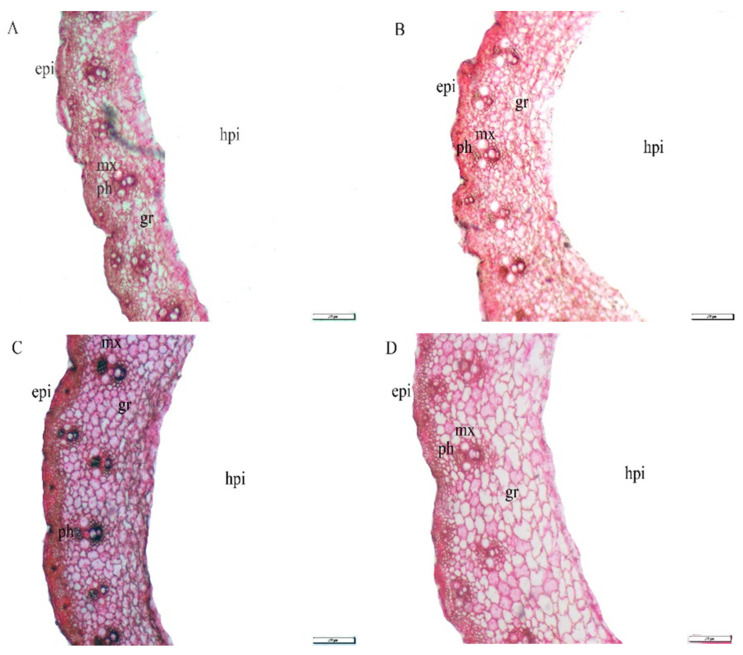
Exogenous application effect of selenium (Se), silicon (Si) and their combinations on stem anatomy of wheat plants grown in saline soil. (**A**) control; (**B**) 30 mM of Se; (**C**) 30 mM of Si; (**D**) 30 mM of Se + Si; (epi = epidermis, mx = metaxylem vessel, ph = phloem, gr = ground tissue and hpi = hollow pith cavity).

Table 2 and Figure 2 represent the changes in the anatomical structure of wheat plants grown under salt stress conditions as well as salt-stressed wheat plants treated with Si, Se and their combinations. Applications of Se or Si as individual applications at 15 and 30 mM and their combinations caused an ameliorative influence on the anatomical traits of wheat stems. Exogenous application of wheat plants with Se_30_ + Si_30_ was the most effective treatment in terms of improving the stem anatomical structure. Compared to the control, the applications of Se_30_ + Si_30_ and Se_15_ + Si_15_ resulted in increases of 73.94 and 66.45% for section diameter, owing to the increase in the diameter of the hollow pith by 67.94 and 57.58%, respectively. In addition, the exogenous application of the combination treatments, i.e., Se_30_ + Si_30_ and Se_15_ + Si_15_, resulted in an increase in ground tissue thickness of 53.85 and 27.69%, ground tissue number of 57.14 and 28.57 as well as increases in the diameter of metaxylem vessels of 41.94 and 29.03%, vascular bundle length of 95.65 and 41.74%, width of 50 and 50%, and number of cell layers of 69.57 and 52.17%, respectively, compared with untreated salt-stressed plants (control).

**Figure 3:**
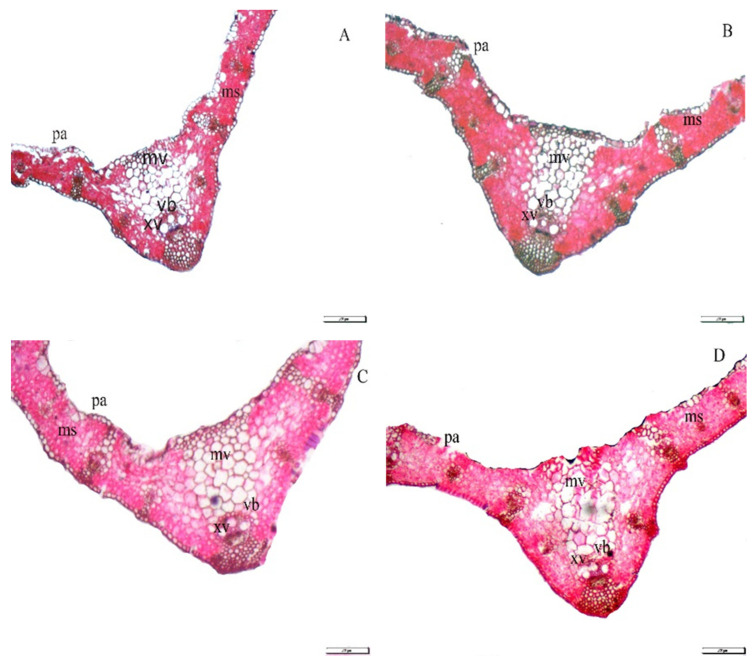
Exogenous application effect of selenium (Se), silicon (Si) and their combinations on leaf anatomy of wheat plants grown in saline soil. (**A**) control; (**B**) 30 mM of Se; (**C**) 30 mM of Si; (**D**) 30 mM of Se + Si; (mv = mid vein, vb = vascular bundle, xv = xylem vessel, pa = Palisade and ms = mesophyll tissue).

In the Table 3 and Figure 3, the application of Se or Si individually or in combination resulted in increases in blade thickness, mesophyll thickness, midvein thickness, mid vascular bundle diameter and metaxylem vessel diameter. The highest values were recorded for the treatments Se_30_ + Si_30_ and Se_15_ + Si_15_ compared to the individual applications of Se and Si as well as untreated plants. The increases as a result of Se_30_ + Si_30_ and Se_15_ + Si_15_ applications were 36.84 and 31.58% for blade thickness, due to increase in mesophyll thickness by 60.00 and 48.00%, respectively, compared with the control. In addition, the increases due to the application of Se_30_ + Si_30_ and Se_15_ + Si_15_ in midvein thickness were 17.67 and 11.67%, related to increases in mid-vascular bundle diameter by 29.6 and 24%, enhancing metaxylem vessel diameter by 33.33 and 20.00%, respectively, compared with the control.

### 3.3. Physiological Attributes of Triticum Aestivum

**Figure 4:**
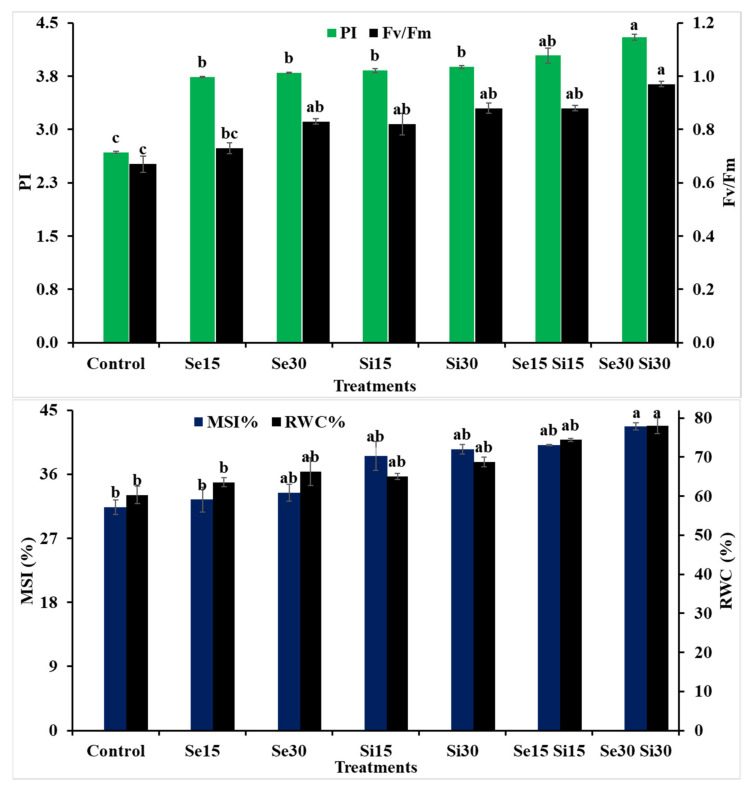
Exogenous application effect of selenium (Se), silicon (Si) and their combinations on physiological attributes of wheat plants grown in saline soil. Columns of each traits with the same letters are not significantly different at *p* ≤ 0.05.

The data in Figure 4 show that the exogenous application of Si, Se and their combinations on wheat plants had a significant effect in ameliorating photosynthetic efficiency indices (i.e., Fv/Fm and PI), relative water content (RWC), and membrane stability index (MSI) compared with the untreated plants grown under salinity stress conditions. The treatment of Si_30_ + Se_30_ was found to be the most effective one since it caused an increment in Fv/Fm by 44.78%, PI by 60.45%, RWC by 29.39% and MSI% by 36.39% compared to the untreated plants.

**Figure 5:**
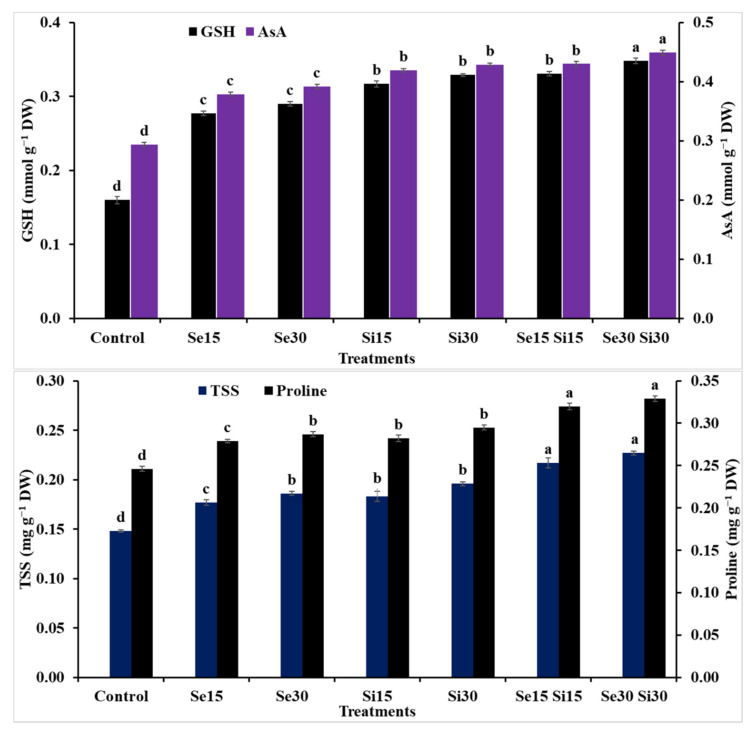
Exogenous application effect of selenium (Se), silicon (Si) and their combinations on contents of non-enzymatic antioxidants and osmoprotectants of wheat plants grown in saline soil. Columns of each traits with the same letters are not significantly different at *p* ≤ 0.05. GSH = glutathione; AsA = ascorbic acid; TSS = total soluble sugars.

**Figure 6:**
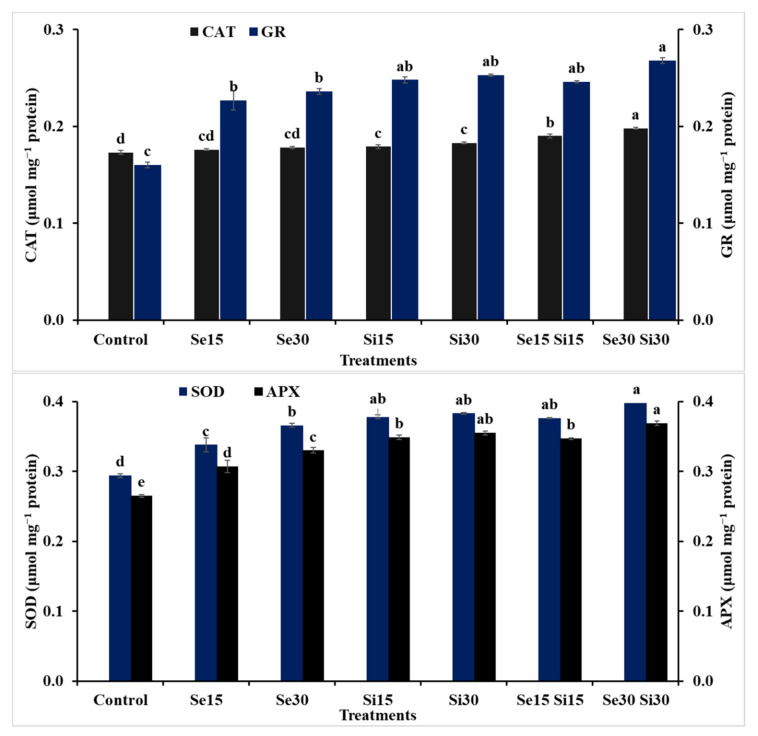
Exogenous application effect of selenium (Se), silicon (Si) and their combinations on the activities of enzymatic antioxidants of wheat plants grown in saline soil. Columns of each traits with the same letters are not significantly different at *p* ≤ 0.05. CAT = catalase; GR = glutathione reductase; SOD = superoxide dismutase; APX = ascorbate peroxidase.

The application of Se, Si and their combinations at different concentrations demonstrated a significant effect on the activity of enzymatic and non-enzymatic antioxidants and osmoprotectants of wheat plants grown under salinity conditions (Figure 5 and Figure 6). The exogenous spray of Si as an individual application at both concentrations (i.e., Si_15_ and Si_30_) showed better results than Se (i.e., Se_15_ and Se_30_) as an individual application. The Si treatment increased TSS content by 23.64 and 32.43%, free proline content by 14.63 and 19.92%, GSH content by 98.13 and 105.63%, AsA content by 42.52 and 45.92%, CAT activity by 3.47 and 5.78%, GR activity by 55 and 58.13%, SOD activity by 28.57 and 30.27% and APX activity by 31.69 and 33.96%, respectively, compared to untreated plants. While the combinations of Si and Se demonstrated the best results, compared to other treatments, the combination treatment between Si and Se at 30 mM showed better values than the combination treatment of Si and Se at 15 mM for GSH, AsA, CAT and APX; there were no significant differences for both combination treatments on proline and TSS. The increases as a result of the combination treatment of Se_30_ + Si_30_ were TSS content by 53.38%, free proline content by 33.74%, GSH content by 117.5%, AsA content by 52.32%, CAT activity by 14.45%, GR activity by 67.5%, SOD activity by 35.37% and APX activity by 39.25%, compared to the untreated plants (control).

### 3.4. Yield and Its Components of Triticum Aestivum

**Figure 7:**
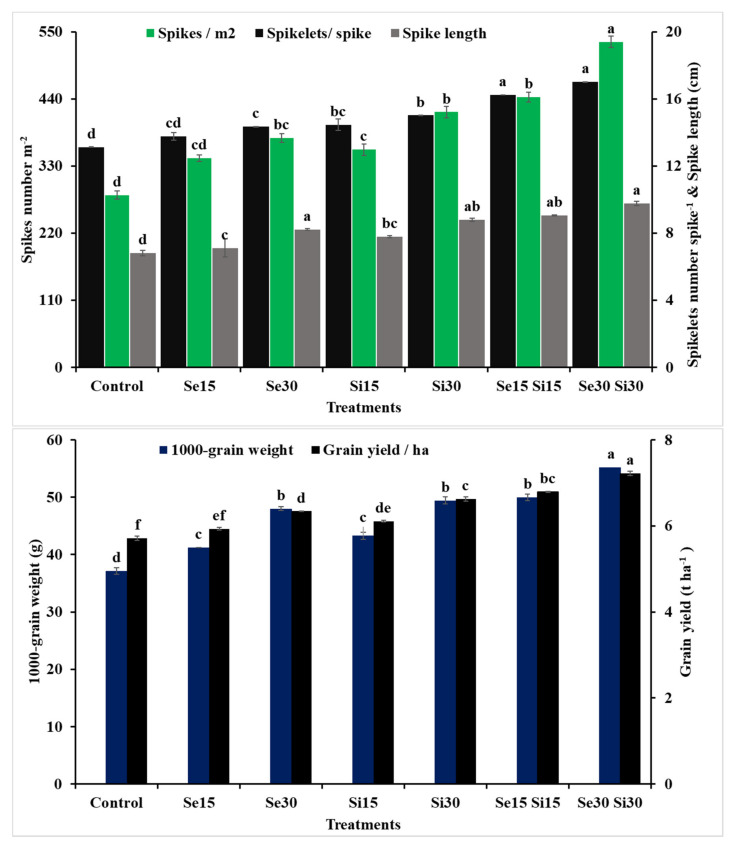
Exogenous application effect of selenium (Se), silicon (Si) and their combinations on yield and components of wheat plants grown in saline soil. Columns of each traits with the same letters are not significantly different at *p* ≤ 0.05.

Wheat yield was positively impacted by the foliar spray of Se, Si and their combinations under field salt stress conditions (Figure 7). The application of Se or Si at different concentrations, except Se_15_ treatment, dramatically improved wheat yield and its components compared to the untreated plants. Moreover, the combined effect of Se_30_ and Si_30_ was the most influential treatment, following by the combination treatment of Se_15_ and Si_15_*,* since both of these caused increases of 88.95 and 56.94% in number of spikes (m^−2^), 29.55 and 23.61% in number of spikelets spike^−1^, 43.47 and 33.03% in spike length, 48.73 and 34.75% in average 1000-grain weight and 26.44 and 18.91% in grain yield (hectare^−1^), respectively, compared to those obtained from the control treatment. The individual applications of Si showed better results in comparison to the individual applications of Se on different grain yield in ha^−1^ and number of spikelets spike^−1^.

## 4. Discussion

Abiotic stresses including salinity can significantly impact plant growth, anatomical features, and physiological and biochemical traits. Consequently, the crop yield can be negatively affected if stress occurs in the sensitive stages of plant life. Salinity stress is considered a main problem, particularly in developing countries where people are more dependent on the agricultural sector. For example, the accumulated salts in the soil solution can cause an osmotic pressure and reduce the available water to the plants [59,60,61,62]. Furthermore, the excessive accumulation of Na^+^ and Cl^−^ can cause an imbalance of ionic and ion toxicity that can reduce the absorbance of other mineral nutrients by plants [14]. Additionally, it can promote the formation of abscisic acid (ABA) and reduce growth promoters [19,63]. Thus, imbalances in ionic and water content of the plant, stomatal closure, and lowered photosynthesis are considered the most prevalent salinity damages [18]. The exogenous application of wheat plants with selenium (Se), silicon (Si) and their combinations improved plant height, number of tillers (m^−2^), and shoot fresh and dry weight plant^−1^ as growth parameters (Figure 1). The Se and/or Si–induced ameliorations in growth traits of wheat might be owing to their function in the regulation of physiological processes, inclusive of photosynthesis [64,65], water status in plant tissue (Figure 4), and machinery of antioxidants [21]. The stimulating role for Se and Si in chlorophyll synthesis, protein, and in absorbance of necessary nutrients was positively observed in wheat growth and productivity (Figure 1 and Figure 7; [66]). Silicon might have a positive role in the metabolic activities of plants exposed to salinity stress [65]. Moreover, it affects plant transpiration rate by silicating the surface of leaves and reducing the stomata lumen [38,67]. Silicon can boost the absorption and translocation of different nutrients [65]. In the same context, the application of Se can lead to the disorder of amino acids metabolism; consequently, it can improve the soluble protein content and reduce the activity of nitrate in plants subjected to stress [68]. Additionally, Se can boost the accumulation of starch in chloroplasts [69] and can protect plant cells [22] and thus can promote plant growth under salt-stressed conditions.

In the current work, the efficiency of wheat photosynthesis, represented in the performance index (PI), Fv/Fm, was significantly reduced under salinity stress. In contrast, Si and Se in individual and combined applications decreased the influence of salt stress and regulated the photosynthetic functions via lowering the production of ROS, which is partially responsible for inhibiting photosynthetic pigments. In addition, protecting the integrity of the chloroplast structure from salinity-induced destruction [70,71] can increase the pigments of chlorophyll and its activity in the enzymes biosynthesizing within the plant tissues under the production of the excessive ROS. Otherwise, Se can regulate numerous physio-biochemical processes, causing an increment in Fv/Fm in chlorophylls, and can activate the antioxidant machinery, which can positively enhance the efficiency of photosynthesis in salinity-stressed plants [72,73]. In the same context, application of Si can ameliorate the activities of several enzymes, boost photosynthesis and improve the water status; it can also prevent the degradation of chlorophyll in plants grown under stress conditions [65].

In the current study, the health of leaf tissue in the untreated plants (control; salinity stress) was negatively affected (i.e., RWC and MSI). This passive result was noted in a salt-stressed leaf because of a transpiration rate higher than the water absorbance. The current investigation demonstrates that exogenous application of Se and/or Si at both concentrations (i.e., 15 and 30 mM) resulted in a significant improvement in the RWC % and MSI %. It is known that salinity can cause a physiological dehydration of plants [14]. However, the exogenous application of Se, Si and their combinations can improve the case of salinity-stressed wheat leaf tissue and can ameliorate their succulency in terms of improving the RWC and MSI. These results could be due to the integrative role of Se and Si in controlling water status when wheat plants are grown under salinity stress [65,74,75]. In addition, Si can increase the leaf thickness and can protect it from transpiration and reduce water losses.

Certainly, Se has a vital function in inhibiting the production of excessive ROS prompted by salinity stress [76]. To conquer ROS overproduction under salinity stress, wheat plants need to raise the internal constituents of their antioxidant defense systems (Figure 5 and Figure 6); this was done via Se. Wheat plants in the current study produced more soluble sugars and proline with supplementation of Se under salinity stress conditions (Figure 5).

Proline and total soluble sugars are organic osmolytes and assist in cell osmoregulation and mitigation of stresses for plants [77,78,79,80]. A close relationship between the concentration of osmoprotectants and tolerance of stress has been noted, and they are considered one of the indicators for the possibility of stress tolerance [81]. Moreover, they can lessen the stress-incurred adverse impacts via regulating important protein formation like Rubisco, protection of the photosynthetic apparatus, amelioration of the membrane stability, maintaining the redox balance, and removing the ROS [82]. When subjected to salinity stress, plants interact with internal Si by increasing metabolite formation through soluble sugars and proline. These metabolites can induce tolerance to salinity stress via the osmotic balance, which maintains the turgor of cells and ensures the constancy and safety of cellular membranes, thus prohibiting or lowering photo-oxidation and inhibiting the oxidative harm. In our study, Se and/or Si application boosted organic compounds such as TSS and proline (Figure 5). The increase in total soluble sugars might be attributed to the enhanced amylase activity and to the hydrolyzing of starch into sugars by Se; it could also be due to fructose 1, 6-bisphosphatase, which plays role in carbohydrate metabolism. Wheat plants exposed to salinity stress exhibited an augmentation of the TSS and proline accumulation in leaf tissue compared to the unstressed plants. These increments in concentrations of proline and TSS in wheat leaves under salinity stress are a mechanism of the acclimation to salinity stress. A further increment in the proline and TSS accumulation was noted from spraying the wheat plants with Se, Si and their combinations. Soil salinity results in an osmotic stress and can lessen water uptake by roots, eventually causing a disturbance in the functions of roots and stomata [18,83,84]. This might be the reason for the reduction of the stomatal conductance in salt-stressed wheat plants. Under salt-stress conditions, organic osmolytes (osmoprotectants) can assist the plant cells in adapting to the osmosis and can establish a gradient of osmosis to regulate the ability of plants’ water uptake [60,61,85]. In the current investigation, an increment in the accumulation of TSS and proline in response to both Se and Si and their combinations could have participated in the mediation of plant water conditions and the hydraulic conductivity during salinity stress. In addition, Se/Si-treated wheat plants had a high proline accumulation, which might have participated in the ROS scavenging to mitigate oxidative harm in the current study.

Plants exposed to salt stress can produce ROS, which can cause oxidative harm and induce oxidative cell death [86]. Plants contain an internal defense system that comprises enzymatic and non-enzymatic antioxidants to mitigate the oxidative destruction of ROS [86]. The present investigation demonstrated that salt stress resulted in wheat plants’ oxidative harm, and this could be due to augmentation of levels of O_2_^−^ and H_2_O_2_, increasing the lipid peroxidation rate in leaves. Wheat plants treated with Se, Si and their combinations avoided salinity stress through activating the antioxidant defense system; thus the salinity-stressed plants exhibited slightly augmented activity of antioxidants in leaves. Exogenously applied Se and Si and their combinations can improve the ROS scavenging system by hastening the activities of antioxidants in salt-stressed wheat plants, thus alleviating the salinity-induced oxidative damage in wheat leaves. In this context, investigations have demonstrated that Se and Si can alleviate the oxidative damage via regulating antioxidant system activities under salinity stress [87,88]. In addition, the improvement in the activity of the antioxidant enzymes could be related to Se and Si as co-agent mediators that can promote enzymatic antioxidant activity. Furthermore, the safeguarding of membranes and improvement of H^+^-ATPases in membrane proteins by Se and Si supplementation might be a cause for reduced oxidative harm [89,90].

In the current investigation, the salinity stress resulted in a reduction in wheat productivity (Figure 7); however, these passive results were improved with the individual application of Se and/or Si. The increment in wheat production might be because of the roles of Se or Si in the maintenance of cell turgor and integrity of the membrane as well as the ameliorating of chlorophyll, which helps wheat plants to increase their yield under salinity conditions, as presented in this work. Furthermore, activated antioxidant machinery in the current investigation might improve mineral uptake and consequently increase the yield and its components. Our results are in line with the findings of [91,92]. The affirmative impact of Se on productivity of the salinity-stressed wheat plant is significant for the human health because of the increased grain content of Se concerning the agricultural production of foods in Se-lacking soils in many areas, including Egypt.

The findings of this work suggest that the application of stimulators such as Se to plants can positively impact the productivity of agricultural foods, including wheat in salinity. Thus, the individual application of Se and/or Si can act as biofortification of salt-stressed wheat plants to promote activities of their defense system against ROS. Moreover, the findings achieved in this work demonstrate that the exogenous application of Si at 30 mM was more effective than Se (Figure 7). All other growth properties (i.e., Pi, Fv/Fm, RWC, MSI, TSS, proline and yield) stimulated by the application of Se or Si are related with the improving of the anatomical structure of the wheat plant. These findings propose that Se or Si as well as their combinations ameliorate the deleterious impacts of salinity on the anatomical structure of wheat. The improvements in anatomical structure confer the opportunity for the nutrients to reach cells and thus ameliorate metabolic processes that promote physiological activities leading to vigorous growth and optimal yield of wheat grown in salt-stressed soil.

## 5. Conclusions

In conclusion, the highest application of Se in combination with Si at 30 mM (Se_30_ + Si_30_) resulted in the optimal stimulation for growth, amelioration of the anatomical structure, enhancement of photosynthetic efficiency, and productivity of salt-stressed wheat, followed the application of Se_15_ + Si_15_. In addition, the total soluble sugars and total proline in plant tissues was increased. However, the individual application of Se or Si demonstrated that Si effects surpassed the effects of Se on wheat grown in saline soil. Thus, the exogenous application of Se or Si can effectively ameliorate salinity stress tolerance by upregulating the activity of enzymatic antioxidants and non-enzymatic antioxidants and osmolytes, which affirmatively reflected the improvement of growth and grain yield of wheat. Finally, exogenous application of Se in combination with Si at rate of 30 mM is considered the most effective treatment for ameliorating the performance of salt-stressed wheat plants.

## Figures and Tables

**Table 1:** Soil analysis of the experimental location during the two seasons *.

Properties	2018/2019	2019/2020
**Physical Analysis:**		
Clay (%)	13.8	14.0
Silt (%)	17.6	18.8
Sand (%)	68.6	67.2
Texture class	Loamy sand	Loamy sand
**Chemical Analysis:**		
pH (1:2.5)	7.38	7.38
EC (dS m^−1^)	7.59	7.63
Organic matter (%)	0.92	0.92
CaCO_3_ (%)	4.78	4.84
Total N (%)	0.065	0.071
Available P (mg kg^−1^ soil)	7.48	7.54
Available K (mg kg^−1^ soil)	173	181
Se K (mg kg^−1^ soil)	0.19	0.21
Si K (mg kg^−1^ soil)	0.52	0.56

* All analyses were done in the Central Laboratory of Soil, Water and Plant Analysis (Iso17025), Faculty of Agriculture, Fayoum University, Egypt.

**Table 2:** Exogenous application effect of selenium (Se), silicon (Si) and their combinations on stem anatomy of wheat plants grown in saline soil.

Treatments	Sclerenchymatous Tissue			Ground Tissue		
	Thickness (μ)	Number of Cell Layers	Diameter of Cells	Thickness (μ)	Number of Cell Layers	Diameter of Cells
Control	38 ^d^	4 ^c^	18 ^c^	325 ^d^	7 ^d^	38 ^d^
Se_15_	39 ^d^	4 ^c^	19 ^c^	400 ^b^	9 ^c^	41 ^c^
Se_30_	40 ^d^	6 ^ab^	20 ^c^	400 ^b^	10 ^b^	43 ^b^
Si_15_	63 ^c^	4 ^c^	19 ^c^	375 ^c^	9 ^c^	41 ^c^
Si_30_	73 ^b^	7 ^a^	23 ^b^	385 ^bc^	10 ^b^	44 ^b^
Se_15_ + Si_15_	75 ^b^	5 ^bc^	25 ^b^	415 ^b^	9 ^c^	50 ^a^
Se_30_ + Si_30_	80 ^a^	7 ^a^	30 ^a^	500 ^a^	11 ^a^	51 ^a^
**Treatments**	**Vascular Bundles**			**Diameter of mx Vessels (μ)**	**Diameter of Hollow Pith (μ)**	**Section Diameter (μ)**
	**Length (μ)**	**Width (μ)**	**Number**			
Control	115 ^e^	100 ^d^	23 ^d^	31 ^b^	1563 ^f^	2313 ^f^
Se_15_	125 ^d^	115 ^c^	27 ^c^	36 ^ab^	1568 ^f^	2700 ^e^
Se_30_	125 ^d^	125 ^b^	29 ^c^	37 ^ab^	1688 ^e^	2800 ^d^
Si_15_	150 ^c^	125 ^b^	28 ^c^	39 ^ab^	1813 ^d^	2875 ^d^
Si_30_	150 ^c^	130 ^b^	30 ^c^	40 ^ab^	2188 ^c^	3213 ^c^
Se_15_ + Si_15_	163 ^b^	150 ^a^	35 ^b^	40 ^ab^	2463 ^b^	3850 ^b^
Se_30_ + Si_30_	225 ^a^	150 ^a^	39 ^a^	44 ^a^	2625 ^a^	4000 ^a^

Mean values in the same column for each trait with the same letters are not significantly different.

**Table 3:** Exogenous application effect of selenium (Se), silicon (Si) and their combinations on leaf anatomy of wheat plants grown in saline soil.

Treatments	Thickness of Mesophyll (μ)	Thickness of Midvein (μ)	Diameter of Mid-Vascular Bundle (μ)	Thickness of Blade (μ)	Metaxylem Diameter (μ)
Control	125 ^f^	600 ^f^	125 ^e^	190 ^c^	30 ^d^
Se_15_	140 ^e^	630 ^e^	130 ^de^	195 ^c^	30 ^d^
Se_30_	158 ^d^	650 ^c^	135 ^d^	220 ^b^	33 ^c^
Si_15_	150 ^d^	640 ^d^	132 ^d^	200 ^b^	32 ^c^
Si_30_	175 ^c^	650 ^c^	140 ^c^	270 ^a^	36 ^b^
Se_15_ + Si_15_	185 ^b^	670 ^b^	155 ^b^	250 ^a^	36 ^b^
Se_30_ + Si_30_	200 ^a^	700 ^a^	165 ^a^	260 ^a^	40 ^a^

Mean values in the same column for each trait with the same letters are not significantly different.

## Data Availability

All data is presented within the article or in Supplementary Materials.

## Supplementary Materials

The following are available online at https://www.mdpi.com/article/10.3390/plants10061040/s1, Table S1: Weather data during the whole course of study at El-Fayoum region, Egypt.
