# Supplementary material for: Integrated Application of Selenium and Silicon Enhances Growth and Anatomical Structure, Antioxidant Defense System and Yield of Wheat Grown in Salt-Stressed Soil
Source: Plants (Basel). 2021 May 21;10(6):1040. doi: 10.3390/plants10061040 (PMC8224300; doi:10.3390/plants10061040)
Supplement: Supplementary file 1 [file plants-10-01040-s001.zip › plants-1234664-supplementary.pdf]

Table S1: Weather data during the whole course of study at El-Fayoum region, Egypt

	Mean temperature (°C)		Mean relative humidity (%)	Precipitation (mm d^{-1})
	Day	Night		
**First growing season (2018–2019)**				
Nov.	28.10	15.60	42.0	0.18
Dec.	21.00	9.50	42.0	0.24
Jan.	20.50	8.50	42.6	0.03
Feb.	22.00	8.50	42.0	0.10
Mar.	28.30	12.60	36.6	0.12
**Second growing season (2019–2020)**				
Nov.	27.90	15.90	41.5	0.23
Dec.	22.10	9.70	43.1	0.04
Jan.	20.50	8.50	43.3	0.33
Feb.	23.70	9.80	41.7	0.17
Mar.	28.10	13.30	35.9	0.04

Source: Fayoum Agricultural Research Station, Fayoum province, Egypt
